# Efficient electrocatalytic CO_2_ reduction to ethylene using cuprous oxide derivatives

**DOI:** 10.3389/fchem.2024.1482168

**Published:** 2024-10-14

**Authors:** Wenfei Dong, Dewen Fu, Zhifeng Zhang, Zhiqiang Wu, Hongjian Zhao, Wangsuo Liu

**Affiliations:** ^1^ Ningxia Key Laboratory of Green Catalytic Materials and Technology, College of Chemistry and Chemical Engineering, Ningxia Normal University, Guyuan, China; ^2^ Department of Chemical and Environmental Engineering, Hetao College, Bayannur, Inner Mongolia, China

**Keywords:** CO_2_ reduction, Cu_2_O, coordination numbers, crystal surface regulation, electro-catalysis

## Abstract

Copper-based materials play a vital role in the electrochemical transformation of CO_2_ into C_2_/C_2+_ compounds. In this study, cross-sectional octahedral Cu_2_O microcrystals were prepared *in situ* on carbon paper electrodes via electrochemical deposition. The morphology and integrity of the exposed crystal surface (111) were meticulously controlled by adjusting the deposition potential, time, and temperature. These cross-sectional octahedral Cu_2_O microcrystals exhibited high electrocatalytic activity for ethylene (C_2_H_4_) production through CO_2_ reduction. In a 0.1 M KHCO_3_ electrolyte, the Faradaic efficiency for C_2_H_4_ reached 42.0% at a potential of −1.376 V vs. RHE. During continuous electrolysis over 10 h, the FE (C_2_H_4_) remained stable around 40%. During electrolysis, the fully exposed (111) crystal faces of Cu_2_O microcrystals are reduced to Cu^0^, which enhances C-C coupling and could serve as the main active sites for catalyzing the conversion of CO_2_ to C_2_H_4_.

## 1 Introduction

As fossil fuels continue to be exploited and used, the rising concentration of carbon dioxide has led to severe environmental issues, capturing significant public attention (Obama, 2017). Carbon capture, zero emission and reuse are considered promising strategies for processing and reducing CO_2_ in the atmosphere (Lu et al., 2014). The rise of renewable energy and its important role in the field of energy has attracted people’s attention. Electrocatalytic CO_2_ reduction (CO_2_RR) is viewed as a dependable approach to address this persistent issue. Renewable energy: Wind energy, solar energy and nuclear energy provide sustainable energy which is the continuous driving force of this strategy to realize the conversion of electrocatalytic carbon dioxide to achieve zero CO_2_ emissions (Hardebeck, 2015; Liu et al., 2016).

The CO_2_RR has various electrochemical products including CO, HCOOH, CH_4_, C_2_H_4_, etc. The conversion of C_1_ (CO, HCOOH) products has reached or even exceeded 90% high Faraday efficiency (Wu et al., 2020; Li et al., 2023; Li et al., 2021), while the conversion of other C_2+_ products with higher utility value does not have a high Faraday efficiency (FE) (De Luna et al., 2019). Among various electrocatalytic products, C_2_H_4_ has been widely used in industrial production, polymer manufacturing, and agricultural production (Loiudice et al., 2016; Ren et al., 2019), The conversion of CO_2_RR to C_2_H_4_ is of great significance to industrial production. Currently, copper-based materials are the sole metal substances capable of transforming carbon dioxide into ethylene and C_2+_ products using electrical energy. Despite Cu-based materials being capable of generating C_2_ and C_3_ products like C_2_H_4_, C_2_H_5_OH, and acetic acid, their low selectivity, high overpotential, low current density, stability, and easily affected catalytic environment prevent them from becoming highly efficient catalysts (De Luna et al., 2019; Asadi et al., 2016). This is mainly because the C_2+_ product requires the coupling of intermediates and the complex electron proton transfer process in the catalysis process, which requires a catalyst with high activity and complex morphology and structure to complete (Fan et al., 2020). The effective use of catalysts and their design play crucial roles in improving the electrocatalytic transformation of CO_2_RR to ethylene. Nevertheless, the selectivity, stability, and energy efficiency of this electrocatalytic procedure require further optimization for broader industrial application.

For the design of copper-based catalysts, the current focus is mainly on heating the copper film and performing oxidation treatment, or further reduction in the process of reuse, etc. These processes are all to increase the roughness and defect degree of the copper surface (Li and Kanan, 2012; Kas et al., 2014). At the same time, in terms of improving the Cu-based catalyst, starting from the size, morphology and exposed crystal faces of the copper-based material, focus on optimizing and improving the performance of the electrocatalyst (Liu et al., 2017; Liu et al., 2022; Hori et al., 2002; Zhang et al., 2018). In the highly selective production of C_2_H_4_, Cu_2_O NPs have better catalytic performance than metallic Cu NPs (Liu et al., 2022; Zhang et al., 2018; Ren et al., 2015; Jung et al., 2019). The recently reported Cu/Cu_2_O catalyst prepared by electrodeposition has 36% FE (C_2_H_4_) (De Luna et al., 2018). This could be due to the fact that low-coordination Cu + ions on the surface enhance C-C coupling, which in turn supports the production of C_2_H_4_ (Jung et al., 2019). Recently, Kas et al. (2014) showed that Cu films derived from Cu_2_O can reduce CO_2_ and convert to ethylene, with FEs as high as 34–39%. The increased production of C_2_H_4_ on these films may be linked to the presence of the (100) Cu facet and defect sites (Kas et al., 2014; Ren et al., 2015; Hori et al., 2003). Thermal desorption studies conducted under ultra-high vacuum conditions revealed significant chemical adsorption of CO on Cu derived from Cu_2_O. It is also suggested that residual CuOx species contribute to the catalytic conversion of CO_2_ to C_2_H_4_ (Verdaguer-Casadevall et al., 2015; Kim et al., 2015). Understanding the impact of crystal faces is crucial for managing the activity and selectivity of electrocatalysts. The crystal surfaces of metallic Cu nanoparticles significantly influence the selectivity and activity in catalytic reactions. Theoretical studies indicate that an efficient catalyst should effectively facilitate the conversion of adsorbed CO protons into CHO or COH, while simultaneously displaying minimal activity for the competing hydrogen evolution reaction (Calle Vallejo and Koper, 2013). Adjusting crystal facets, particularly designing high-index crystal facets which possess numerous atomic steps, edges, and unsaturated coordination sites, offers greater potential for developing catalysts with enhanced activity and selectivity compared to merely controlling particle size (Tian et al., 2007; Zhao et al., 2018). The truncated octahedral Cu_2_O nanoparticles, which include both (111) and (100) surfaces, exhibit increased selectivity towards ethylene due to a synergistic interaction among the various low-index surfaces (Gao et al., 2020). Nevertheless, there is limited research exploring the connection between the high-index surfaces of Cu-based catalysts and their CO_2_RR performance (Fan et al., 2020; Gu et al., 2018). Research indicates that Cu_2_O nanoparticles (NPs) with various crystal facets exhibit distinct stability and catalytic behaviors (Jiang et al., 2018; Qin et al., 2019). During the reduction phase involving Cu_2_O, metallic Cu nanoparticles (NPs) develop on the Cu_2_O surface. It remains uncertain if these metallic Cu NPs that form on the Cu_2_O surface serve as active catalysts in the CO_2_RR process (Wang et al., 2016; Lee et al., 2015). The Cu nanoparticles (NPs) derived from various types of Cu_2_O NPs exhibit differences in size and aggregation, impacting the selectivity and activity involved in C_2_H_4_ production (Li and Kanan, 2012). These findings led us to investigate how crystal planes affect the activity of C_2_H_4_ formation from metallic Cu NPs derived from Cu_2_O NPs, and to further examine whether the selectivity and activity of CO_2_RR are influenced by Cu_2_O or metallic Cu NPs.

In this work, we successfully synthesized cross-sectional octahedral Cu_2_O by electrochemical deposition, and explored the potential factors of copper nanosheets derived from Cu_2_O nanoparticles in electrocatalytic CO_2_RR conversion to ethylene. We observed that the existence of different octahedrons on the cross-section of Cu_2_O nanoparticles has a great difference in the catalytic carbon dioxide reduction of the derived copper nanosheets. Simultaneously, we examined the ethylene selectivity and activity associated with the exposed crystal surfaces. Our findings indicate that the exposure of crystal facets during the transformation of octahedral Cu_2_O NPs into copper nanosheets plays a critical role in influencing the catalytic conversion of CO_2_RR to ethylene. Furthermore, our studies clearly demonstrate that metallic Cu NPs, compared to Cu_2_O NPs, have a greater impact on the selectivity and activity of C_2_H_4_. Copper nanosheets derived from Cu_2_O NPs are the active species for electrocatalytic CO_2_RR. For truncated octahedral Cu_2_O NPs, the display of crystal planes is crucial for revealing the active material in derivatized copper nanosheets. The selectivity of CO_2_ reduction, particularly towards C_2_H_4_, is strongly linked to the exposed crystal facets of Cu particles originating from Cu_2_O.

## 2 Experiment

### 2.1 Materials and reagents

Copper nitrate (Cu (NO_3_)_2_·3H_2_O, ≥99.5% pure) was sourced from Beijing Chemical Plant of China Reagent, while sodium acetate (C_2_H_3_NaO_2_, ≥99.0% pure) and sodium hydroxide (NaOH, 99%) were obtained from Macklin Reagent Network. Acetic acid (CH_3_COOH, ≥99.5% pure) and potassium bicarbonate (KHCO_3_, ≥99.5% pure) were procured from Sinopharm Chemical Reagent Co., Ltd. No additional purification of these chemicals is required. The deionized water used (18.24 MΩ cm) was produced by our laboratory’s ultra-pure water system.

### 2.2 Synthesis of catalyst

The electrolytic cell and electrodes used are as follows: a standard three-electrode device, the constant potential method is used for electrodeposition on the workstation of the electrochemical system (CH 760E, CH Instruments, China). The working electrode is carbon paper (0.5 cm^2^, Toray TGP-H-060), using AgCl or Ag/Ag^+^ electrode and platinum sheet as reference electrode and counter electrode.

The electroplating solution is an aqueous solution composed of 0.02 M (Cu (NO_3_)_2_·3H_2_O and 0.12 M acetic acid buffer solution, and the pH is adjusted to about 5.0 with sodium hydroxide. Electrodeposition is electrolysis in an H-type double-layer constant temperature water bath The Cu_2_O electrocatalyst was synthesized by constant potential method under 70°C water circulation and recorded as 0.02–1,500 (0.02-represents the potential. 1,500 represents the settling time). Each time the deposited Cu_2_O carbon paper sheet, use deionized water thoroughly Clean and blow dry with nitrogen.

### 2.3 Equipment

The sample’s crystal structure was analyzed using an X-ray diffractometer (Smart Lab, Japan) with intelligent target rotation capability. Surface morphology of each electrocatalyst was examined using a cold field emission scanning electron microscope (F-SEM, Regulus 8100, Japan) and high-resolution transmission electron microscopy (HRTEM, JEM-2100uHR, Japan). Elemental analysis was performed with X-ray photoelectron spectroscopy (XPS, Thermo Scientific K-Alpha, US), employing a monochromatic AlKa radiation source at 1,486.6 eV. All the spectral data were acquired in standard environmental conditions.

### 2.4 Electrochemical test

Electrocatalysis is conducted using a standard H-type electrolysis cell that has three electrodes linked to an electrochemical workstation (CH 760E, CH Instrument, China). The device features a cathode and an anode compartment divided by a proton exchange membrane (Nafion 130). Each section holds 30 mL of 0.1 M KHCO_3_ as the electrolyte solution. Reference electrodes and counter electrodes were comprised of AgCl or Ag/Ag^+^ electrodes and platinum sheets, respectively. A self-fabricated electrode was employed as the working electrode. Following this, the products of the electrocatalytic reduction process were analyzed over 30 min via a chronocurrent technique. All electrical potentials noted in this experiment are calibrated against the Ag/AgCl reference electrode as E (VS RHE) = E (VS Ag/AgCl) + 0.222 V + 0.0591 × pH.

### 2.5 Product detection

Prior to conducting the experiment, the cathode chamber was set up with an online trace gas detection system for CO_2_ reduction using gas chromatography (GC) (GC7900, Tianmei, China). This system includes a thermal conductivity detector (TCD) and a flame ionization detector (FID), with nitrogen serving as the carrier gas to analyze and quantify the resultant products. The electrolyte within the cathode chamber was saturated with N_2_ or CO_2_ at a flow rate of 30 mL min^−1^ for no less than 30 min. Concurrent magnetic stirring at 600 rpm during the process ensured thorough mixing of the electrolyte. Linear sweep voltammetry (LSV) recordings were taken at a scan rate of 10 mV s^−1^. Electrochemical surface area (ECSA) was derived from cyclic voltammograms at varying scan rates (5, 10, 20, 40, 60, 80, 100, and 120 mV s^−1^), within potential window from −0.116 to −0.216 vs. RHE. Electrochemical impedance spectroscopy was conducted in a frequency range of 1 MHz to 10^–2^ Hz under open circuit potential.

Then the quantitative gas products were analyzed for at least 30 min at each potential during the CO_2_ electroreduction process. Based on the GC analysis, the current density and FE of the product were determined. The liquid product underwent further analysis. The FE for CO is calculated using the formula below:

$$\text{FE}=\frac{\text{NnF}}{Q}\times 100\%$$

Here, (N) represents the number of electrons needed to synthesize the product, which equals 2 for C_2_H_4_. The variable (n) stands for the total moles of C_2_H_4_ as measured by GC, (F) is the Faraday constant (96,485°C mol^−1^), and (Q) denotes the total accumulated electric charge. These details are recorded using ChemStation.

## 3 Results and discussion

### 3.1 Physical characteristics of nanomicrocrystals

**FIGURE 1:**
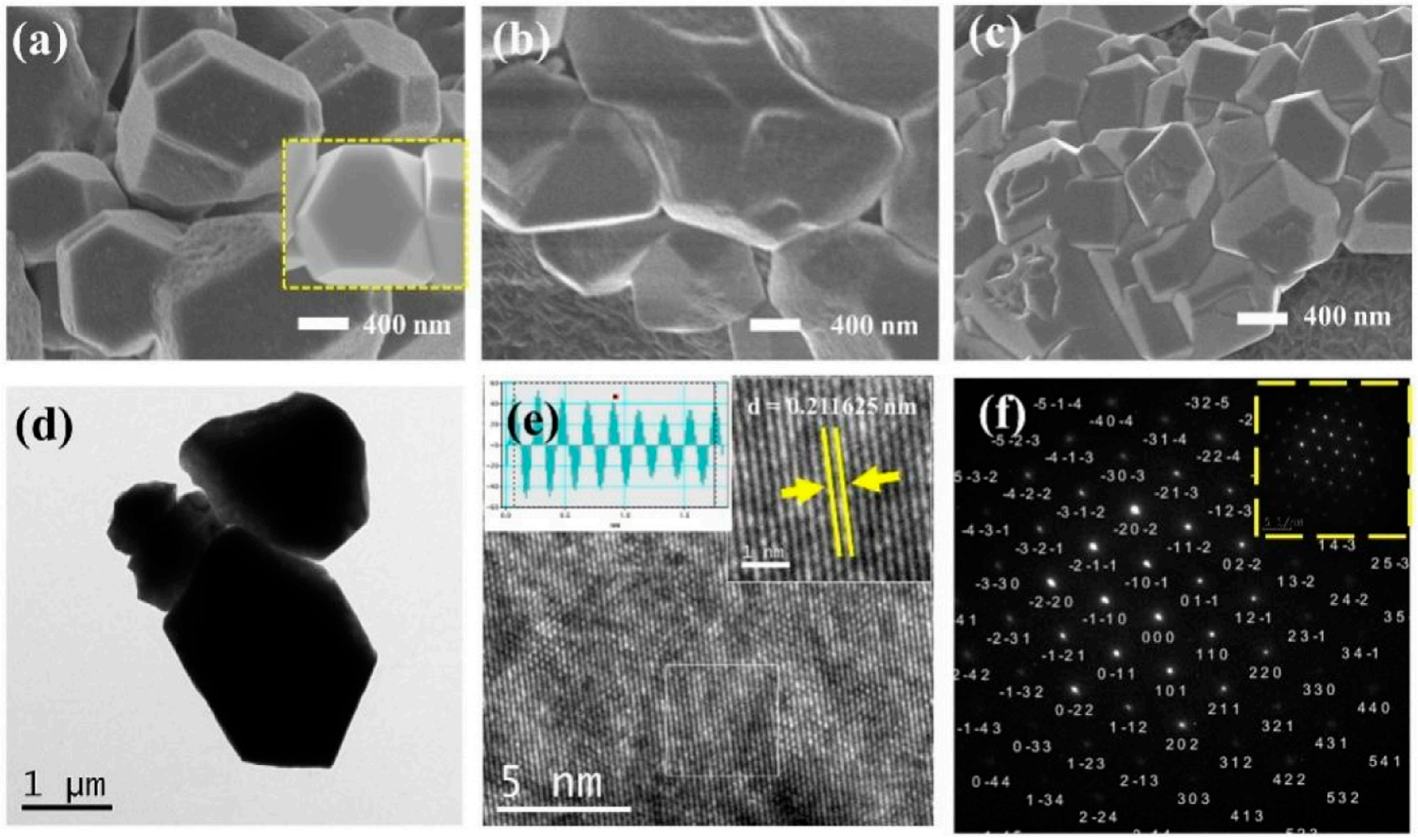
SEM images of Cu_2_O (0.02–1,500) at different electrodeposition temperatures **(A)** 70°C, **(B)** 60°C and **(C)** 80°C; Cu_2_O (0.02–1,500) (70°C) **(D)** TEM images, **(E)** HRTEM images, **(F)** SAED images.

**FIGURE 2:**
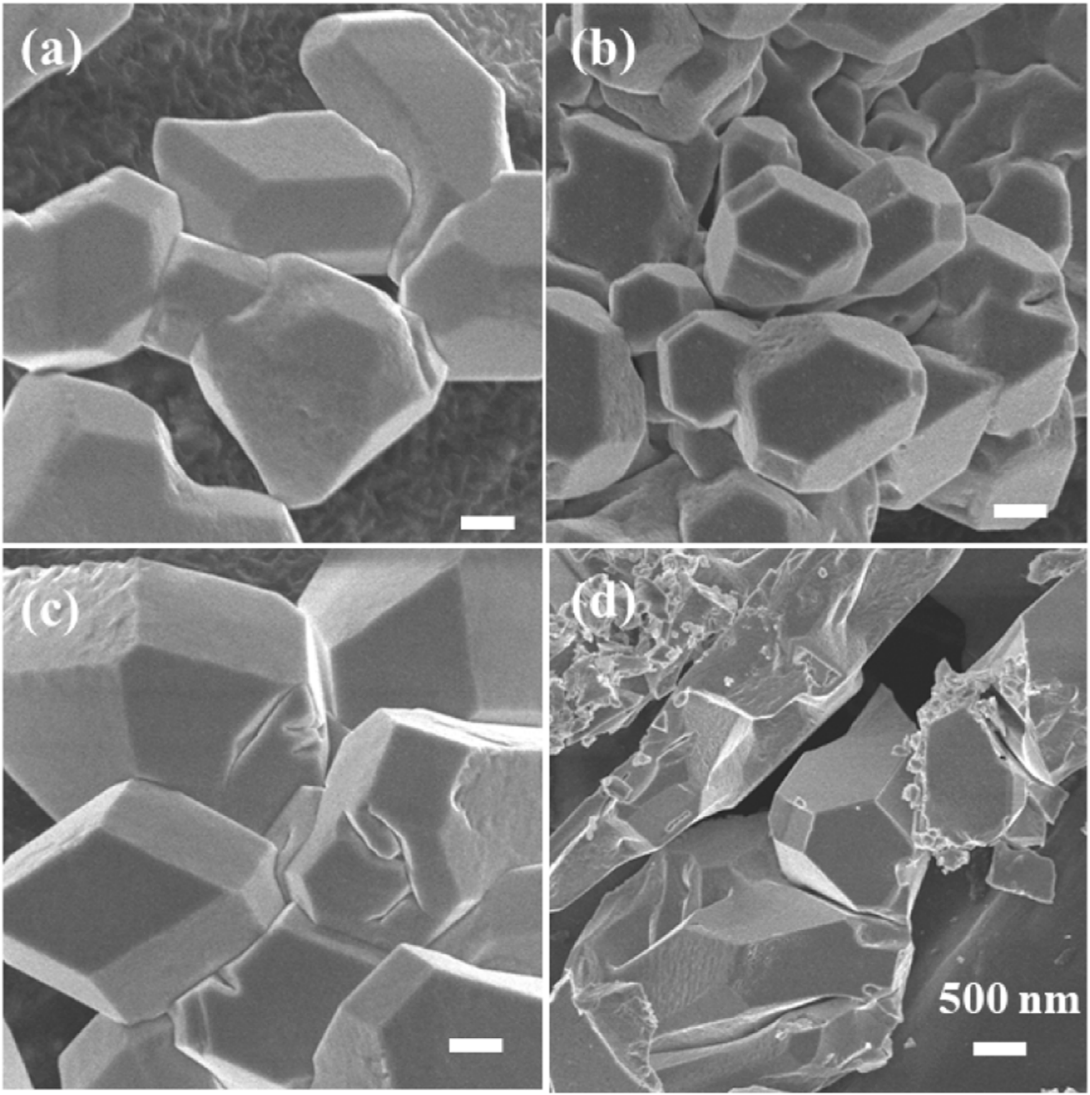
SEM images of Cu_2_O (0.02–70) at different electrodeposition times, **(A)** 1,300 s, **(B)** 1,500 s, **(C)** 1,800 s and **(D)** 3,600 s.

A straightforward constant potential electrochemical deposition technique was utilized to effectively cultivate Cu_2_O particles directly on carbon paper (CP), serving as electrodes with a carbon base. A self-supported electrode like Cu_2_O/CP was prepared. The electrodeposition was carried out in an H-type double-layer constant-temperature water-bath electrolyzer, and the Cu_2_O electrocatalyst was synthesized using the constant-potential method under water circulation at 70°C notated as Cu_2_O 0.02–1,500 (70°C) (0.02- represents the electrodeposition potential, 1,500 represents the deposition time, and 70°C represents the electrodeposition temperature) as shown in Figure 1A. The Cu_2_O microcrystals on the carbon paper (CP) surface were uniformly distributed and have a polycrystalline octahedral morphology (shown in the inset of Figure 1A) with typical (111) and (100) crystal faces. Electrodeposition was performed at temperatures of 60°C and 80°C to establish comparative conditions, with SEM images of the resulting materials presented in Figures 1B, C. The materials electrodeposited at 60°C are specifically depicted in Figures 1B, C. The Cu_2_O microcrystalline particles deposited at 60°C are uniformly distributed, but do not have a complete octahedral morphology, and the Cu_2_O microcrystalline particles obtained by deposition at 80°C are piled up together, and the crystalline faces of the cross-sectional octahedra are incompletely exposed, with only some of the crystalline faces being exposed and the other crystalline faces interspersed with each other to hide them. Comparison of the electrodeposition temperatures reveals that the Cu_2_O microcrystals are uniformly distributed and the crystal faces are well exposed at 70°C. To further explore the microstructural characteristics, TEM images of the Cu_2_O catalyst are displayed in Figure 1D, while the HRTEM and SAED (selected area electron diffraction) images are illustrated in Figures 1E, F. The TEM images illustrate clearly defined crystal faces of Cu_2_O particles. The marked lattice stripe distance d of 0.212 nm aligns with the crystal face spacing of Cu_2_O, and the SAED patterns observed highlight the (111) and (100) crystal planes of the octahedral cross-section of Cu_2_O. Figure 2 shows the SEM images of Cu_2_O (0.02–70) at different electrodeposition times, demonstrating the effect of electrodeposition time on the morphology, and determining that 1,500 scan electrodeposit Cu_2_O with a more complete crystal surface.

**FIGURE 3:**
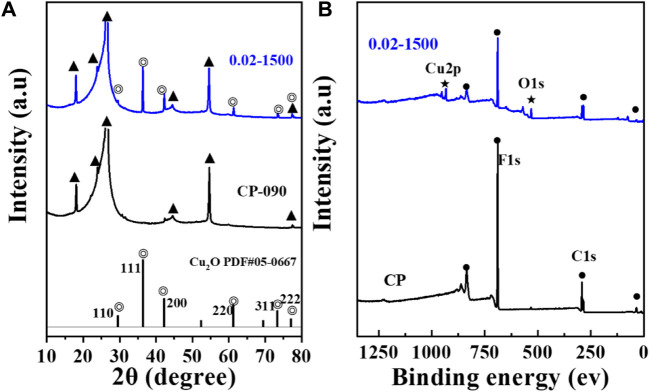
**(A)** XRD pattern of Cu_2_O (0.02–1,500) and CP, **(B)** XPS spectra of Cu_2_O (0.02–1,500) and CP.

Figure 3A presents the X-ray diffraction (XRD) pattern. The figure indicates that the Cu_2_O microcrystals, electrodeposited directly onto the CP substrate, did not fully coat the surface of the carbon paper, and the XRD signal peaks of the carbon paper were observable in the XRD spectrum (▲: denotes the CP diffraction peaks). The main signal peaks of the Cu_2_O crystal structure (◎: denotes the Cu_2_O signal peaks) were in agreement with the standard spectrum (Cu_2_O: JCPDS #05-0667) against (Liu et al., 2021). The diffraction peaks of Cu_2_O microcrystals at 29.554 eV, 36.418 eV, 42.297 eV, 61.344 eV, 73.526 eV, and 77.323 eV were attributed to the (110), (111), (200), (220), (311), and (222) crystallographic facets of Cu_2_O, respectively. It indicates that the Cu_2_O catalyst has good crystallinity and structural characteristics of polycrystalline facets.

**FIGURE 4:**
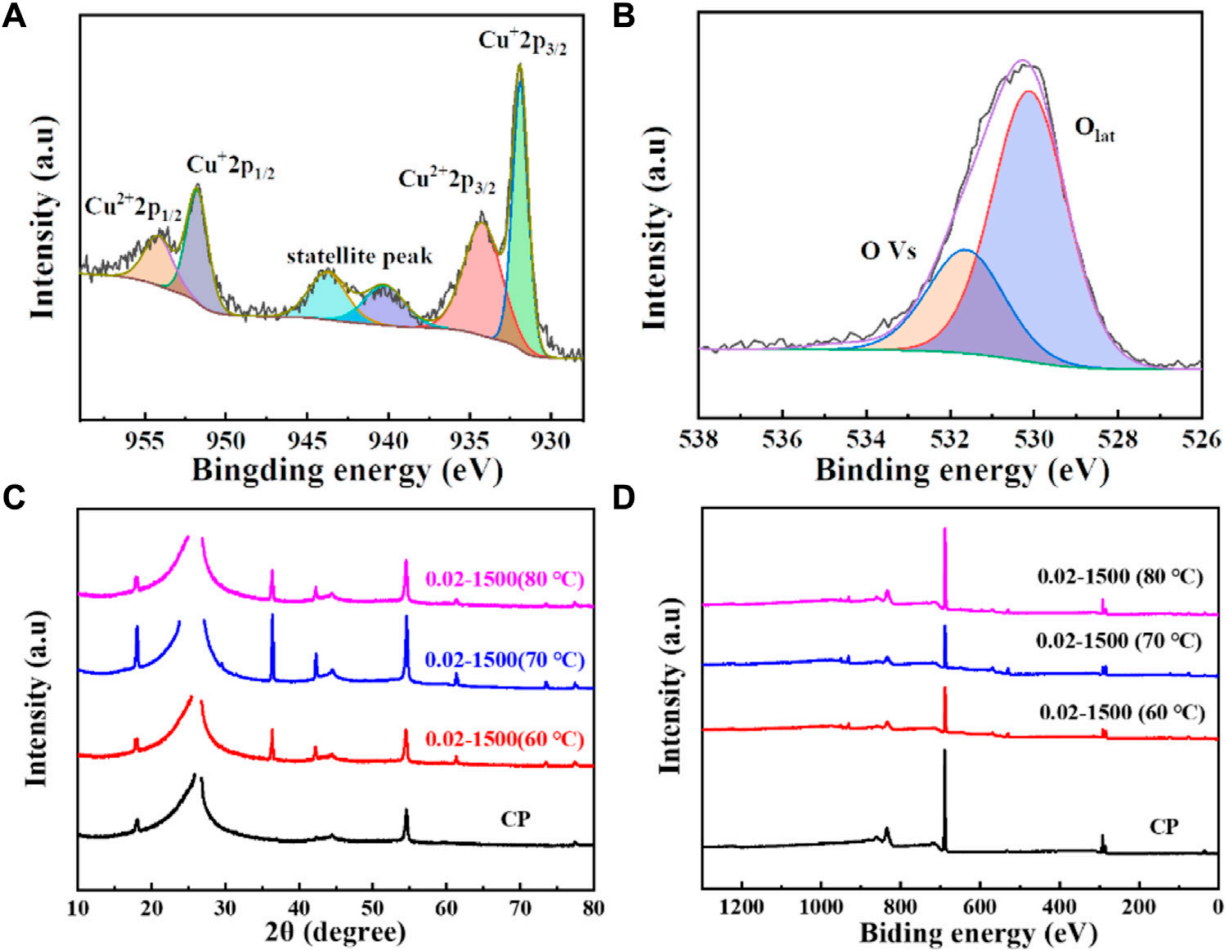
**(A)** Cu 2p spectrum of Cu_2_O (0.02–1,500) (70°C); **(B)** O1 s spectrum of Cu_2_O (0.02–1,500) (70°C); **(C)** XRD pattern of Cu_2_O; **(D)** XPS spectra of Cu_2_O.

**FIGURE 5:**
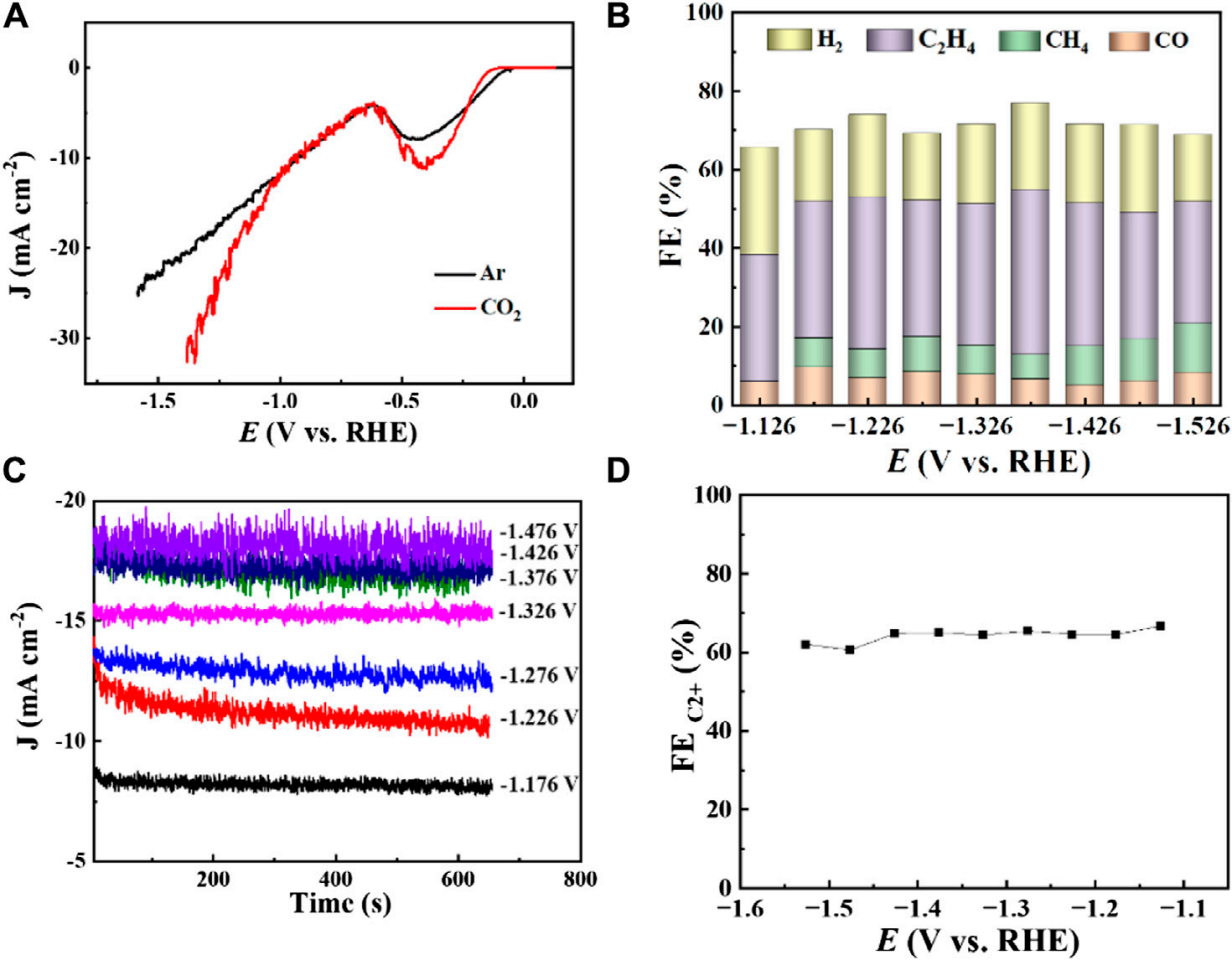
Cu_2_O 0.02–1,500 (70°C) **(A)** LSV curves at CO_2_ and Ar saturated 0.1 M KHCO_3_ with a scan rate of 10 mV s^−1^, **(B)** FE of CO_2_ reduction of different products at different potentials, **(C)** Total current density at different reduction potentials, **(D)** FE of CO_2_ reduction products C_2+_ at different potentials.

The surface valence states of the catalyst were examined using X-ray photoelectron spectroscopy (XPS). As depicted in Figures 3B, 4A, the Cu_2_O microcrystals electrodeposited *in situ* with CP as the substrate contain characteristic peaks of Cu 2p and O 1s, as well as information on the elements contained in the substrate carbon paper. The characteristic peaks with binding energies of 931.88 eV and 951.78 eV are attributed to Cu^+^ 2p^3/2^ and Cu^+^ 2p^1/2^, which can be categorized as (Cu^+^) of Cu_2_O. The predominant Cu^2+^ 2p^3/2^ and Cu^2+^ 2p^1/2^ features at 934.28 eV and 954.08 eV can be attributed to the presence of (Cu^2+^) or a small amount of CuO in Cu_2_O. Satellite peaks appear in the binding energy range of 945 eV–940 eV, indicating that Cu(I) is the primary valence state of the copper species. The presence of Cu (II) results from the oxidation of Cu(I), as Cu_2_O is thermodynamically unstable under typical conditions. Figures 4C, D It can be determined that Cu_2_O obtained at different deposition temperatures: 0.02–1,500 (60°C), 0.02–1,500 (70°C), 0.02–1,500 (80°C) are homogeneous compounds, and by comparing the SEM as in Figures 1–5 a great difference in morphology is found.

### 3.2 Electrochemical CO_2_ reduction properties of Cu_2_O microcrystals

The electrocatalytic CO_2_ reduction performance of Cu_2_O 0.02–1,500 (70°C) was evaluated as illustrated in Figure 5A. This catalyst was tested in a 0.1 M KHCO_3_ solution saturated with both CO_2_ and Ar, where it exhibited a significant reduction peak from −0.25 V to −0.5 V (vs. RHE), likely due to the inherent electrochemical reduction properties of Cu_2_O. Additionally, the intensity of these reduction peaks was higher in the CO_2_-saturated environment compared to the Ar-saturated one, indicating more pronounced reduction activities in the presence of CO_2_. This enhanced peak is believed to result from the elevated CO_2_ concentration within the CO_2_-saturated medium versus the Ar-saturated solution. Across a broad potential range, the current density of the catalyst Cu_2_O 0.02–1,500 (70°C) in the CO_2_-enriched 0.1 M KHCO_3_ solution surpassed that in its Ar-saturated counterpart, demonstrating the robust catalytic reduction capabilities of Cu_2_O microcrystals in CO_2_-rich environments.

The FE of various gaseous products (H_2_, CO, CH_4_, C_2_H_4_) produced by CO_2_ reduction using Cu_2_O 0.02–1,500 (70°C) were evaluated at different potentials in a 0.1 M KHCO_3_ electrolyte, as shown in Figure 5B. The highest FE, reaching 42%, was observed for C_2_H_4_ at a potential of −1.376 V (vs. RHE) with a total current density of 17 mA cm^−2^, which suggests strong selectivity of the catalyst towards C_2_H_4_ production. Figure 5C illustrates the distribution of total current density across various potentials, indicating an increase in total current density with higher reduction potentials. Figure 5D presents the overall FE of the CO_2_ reduction product C_2+_ at various potentials for the 0.02–1,500 (70°C) catalysts under a 0.1 M KHCO_3_ electrolyte setting, where the FE for C_2+_ products was approximately 60% across all tested potentials. In conclusion, C_2_H_4_ is identified as the primary product of CO_2_ electroreduction, indicating that the Cu_2_O microcrystalline particles have good active sites for the generation of C_2+_ during the CO_2_ electroreduction process. The Cu_2_O 0.02–1,500 (70°C) catalysts achieved 42.0% FE (C_2_H_4_) and more than 60% FE (C_2+_). Table 1 summarizes the FE of copper-based catalysts for C_2_H_4_ production. The comparison reveals that Cu_2_O microcrystalline particles prepared *in situ* by electrodeposition are one of the more desirable catalysts for ethylene production by electrocatalytic reduction of CO_2_.

**TABLE 1:** Performance of different copper-based catalysts for C_2_H_4_ formation via electrochemical CO_2_ reduction.

Catalyst	Electrolyte	Potential	Product	FE (%)	References
Cu_2_O nanocubes	0.1 M KHCO_3_	−1.15 V vs. RHE	C_2_H_4_	31.1	Wang et al. (2019)
Graphene/ZnO/Cu_2_O	0.5 M NaHCO_3_	−0.9 V vs. Ag/AgCl	n-propanol	30	Geioushy et al. (2017a)
Cu_2_O/Cu@NC	0.1 M KHCO_3_	−0.68 V vs. RHE	HCOOH	70.5	Li et al. (2020)
Cu@Cu_2_O	0.1 M KHCO_3_	−1.0 V vs. RHE	C2 = C2 (ethylene and ethanol)	50	Shang et al. (2019)
Cu GNC-VL	0.5 M KHCO_3_	−0.87 V vs. RHE	Ethanol	70.52	Zhang et al. (2019)
In-doped Cu@Cu_2_O	0.1 M KHCO_3_	−0.8 V vs. RHE	CO	87.6 ± 2.2	Wang et al. (2020)
cubic Cu_2_O (c-Cu_2_O) NPs with facets	0.5 M KHCO_3_	−1.2 V vs. RHE	C_2_H_4_	38	Gao et al. (2020)
octahedral Cu_2_O (o-Cu_2_O) NPs	0.5 M KHCO_3_	−1.1 V vs. RHE	C_2_H_4_	45	Robb (2021)
truncated-octahedral Cu_2_O (t-Cu_2_O) NPs with both and (100) facets	0.5 M KHCO_3_	−1.1 V vs. RHE	C_2_H_4_	59	Robb (2021)
Cu_2_O-BDD	0.1 M NaCl	−1.5 V vs. RHE	C_2_H_4_	68.2	Denala et al. (2019)
Cuv-Cu_2_O catalyst	0.1 M KHCO_3_	−0.76 V vs. RHE	C_2_H_4_	51.0	Ren et al. (2020)
Cu_2_O (o-Cu_2_O) NCs	1.0 M KCl	−1.1 V vs. RHE	C_2+_	48.3	Fu et al. (2020)
Hollow Cubic Cu_2_O@Au	0.1 M KHCO_3_	−1.0 V vs. RHE	CO	30.1	Tan et al. (2019)
Cu_2_O-derived Cu catalysts	0.1 M KHCO_3_	−0.98 V vs. RHE	C_2_H_4_	42.6	Handoko et al. (2016)
Cu_2_O/CuO	0.5 M KHCO_3_, 10 mM pyridine and HCl (pH = 5)	−1.3 V vs. RHE	CH_3_OH	6.46	Roy et al. (2020)
GN/Cu_2_O	0.5 M NaHCO_3_	−0.9 V vs. Ag/AgCl	C_2_H_5_OH	9.93	Geioushy et al. (2017b)
ZnO@4Cu_2_O	1 M KOH	−1.0 V vs. RHE	C_2_H_4_	35.5	Zhu et al. (2021)
Cu/Cu_2_O-Ag-x)	1 M KOH	200 mA cm−^2^	C_2+_	60.9	Su et al. (2021)
Cu/Cu_2_O@NG	0.2 M KI	−1.9 V vs. RHE	C_2_-C_3_	56	Zhi et al. (2021)
AuxCu_2_O	0.1 M KHCO_3_	−1.3 V vs. RHE	C_2_H_4_	24.4	Cao et al. (2021)

**FIGURE 6:**
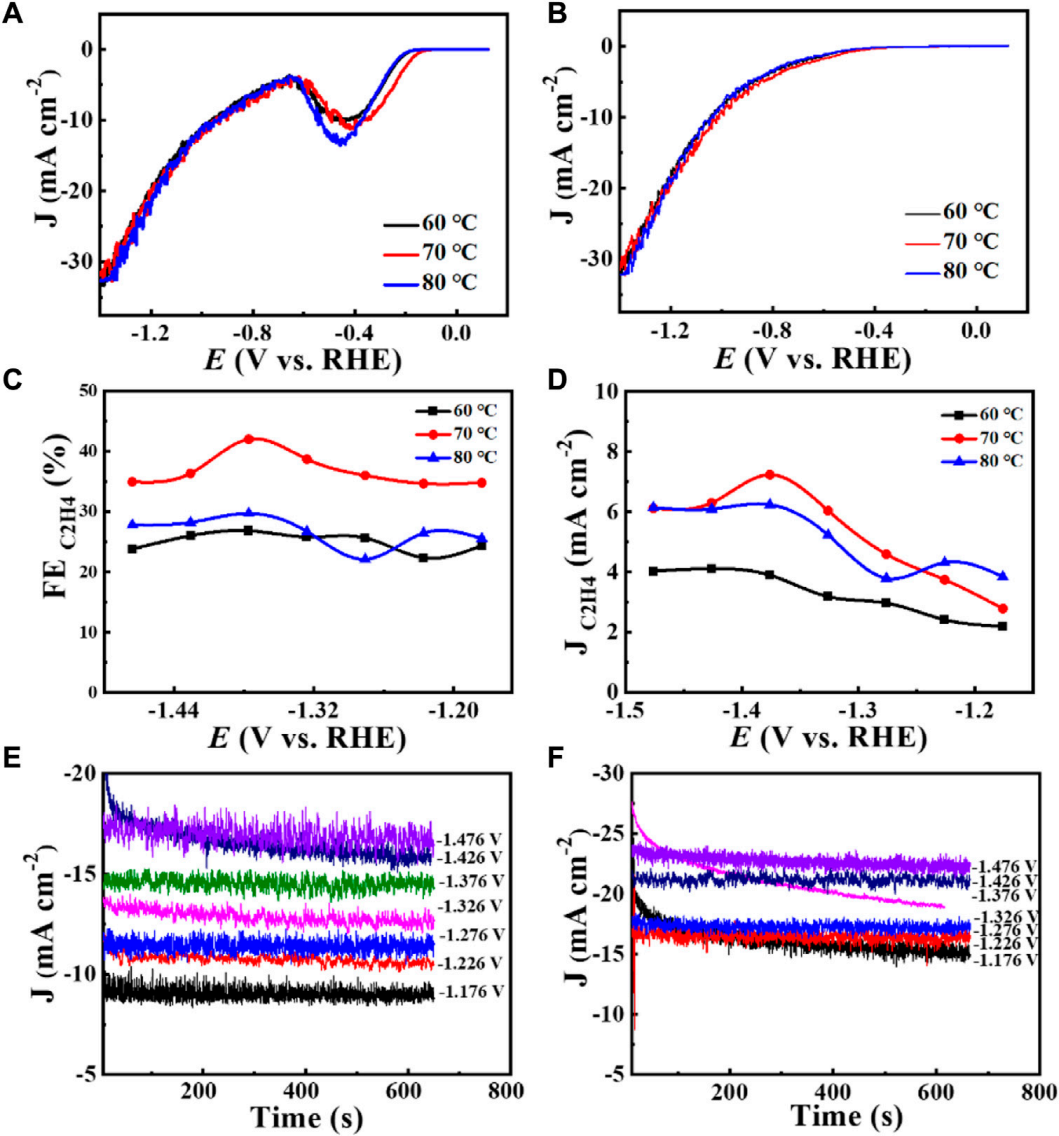
Catalyst Cu_2_O 0.02–1,500 (60°C, 70°C, 80°C): **(A)** 1st LSV curve at CO_2_ and Ar saturated 0.1 M KHCO_3_ with a scan rate of 10 mV s^−1^, **(B)** 2nd LSV curve at CO_2_ and Ar saturated 0.1 M KHCO_3_ with a scan rate of 10 mV s^−1^, **(C)** FE (C_2_H_4_) values as a function of potential, **(D)** J (C_2_H_4_) values as a function of potential, **(E)** Total current density at different reduction potentials from 0.02 to 1,500 (60°C), **(F)** Total current density at different reduction potentials for Cu_2_O 0.02–1,500 (80°C).

**FIGURE 7:**
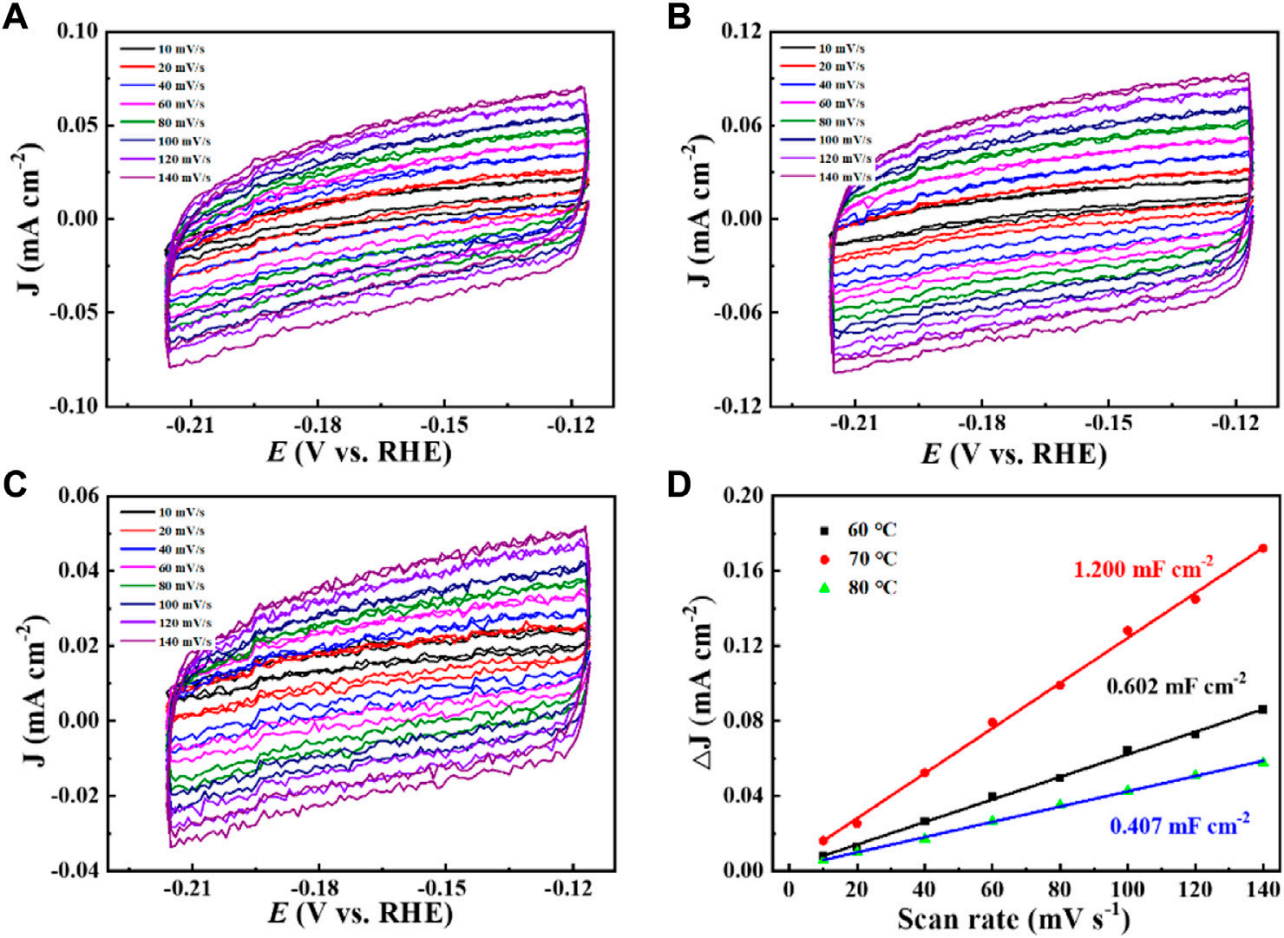
The CVs of **(A)** Cu_2_O 0.02–1,500 (60°C), **(B)** Cu_2_O 0.02–1,500 (70°C), and **(C)** Cu_2_O 0.02–1,500 (80°C), **(D)** Bilayer charge current densities of Cu_2_O 0.02–1,500 (60°C), Cu_2_O 0.02–1,500 (70°C), and Cu_2_O 0.02–1,500 (80°C) versus scan rate.

**FIGURE 8:**
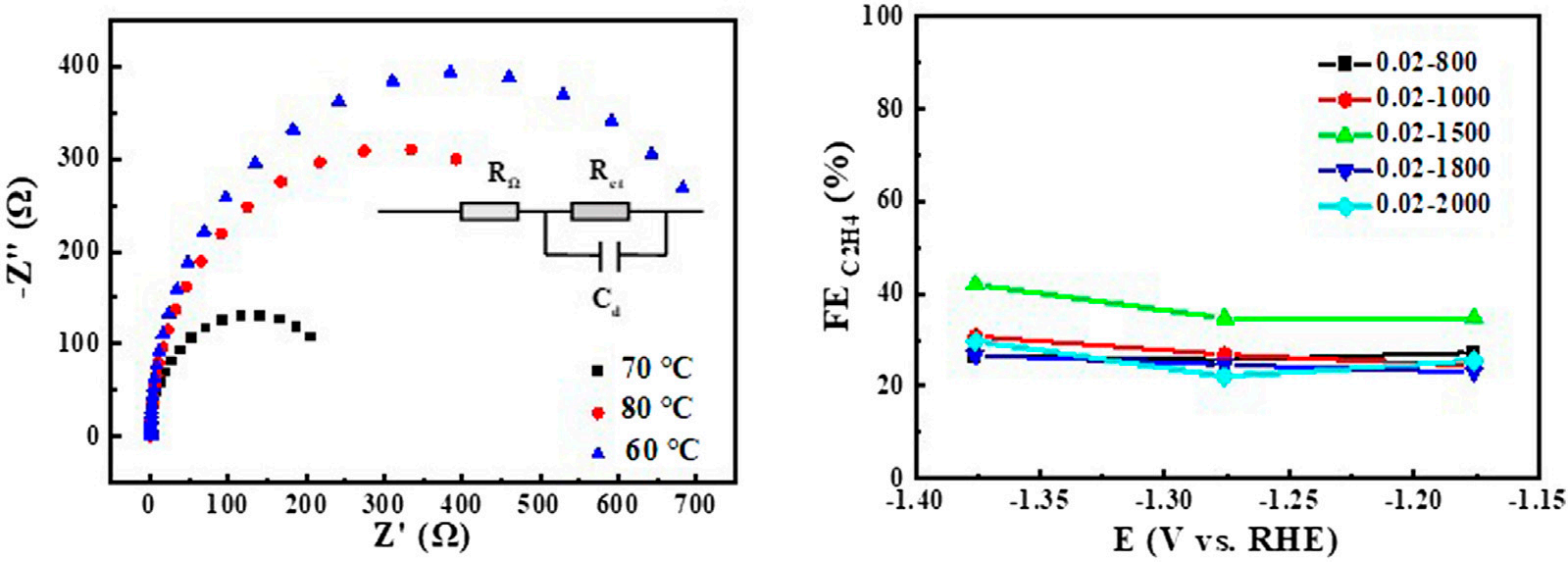
EIS of Cu_2_O 0.02–1500 (60°C, 70°C 0, 80°C) at open circuit voltage for CO_2_ saturated 0.1 M KHCO_3_, relationship between FE of the product ethylene and the potential of CO_2_ reduction for Cu_2_O.

**FIGURE 9:**
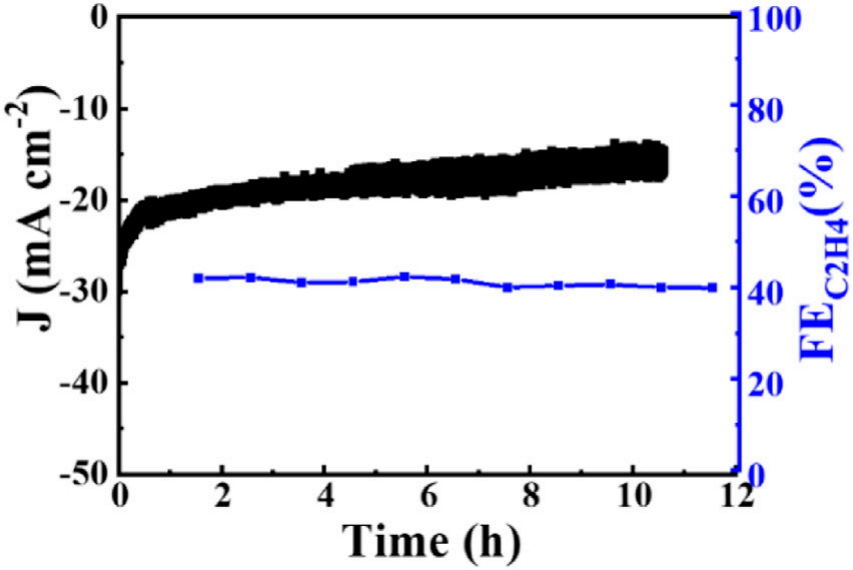
The stability of Cu_2_O 0.02–1,500 (70°C) Cu_2_O in 0.1 M KHCO_3_ electrolyte at −1.376 V (vs. RHE).

Increased current density in a CO_2_-saturated electrolyte suggested an electrochemical CO_2_ reduction reaction (CO_2_RR). Analysis using gas chromatography (GC) revealed the production of C_2_H_4_, CH_4_, CO, and H_2_. The catalyst 0.02–1,500 (70°C) demonstrated significant selectivity towards C_2_H_4_, achieving a faradaic efficiency (FE) of 42.0% at a current density of 7.3 mA cm^−2^ and a potential of −1.376 V (vs. RHE), as shown in Figure 6. The measurement of FE (C_2_H_4_) was repeated three times to obtain a FE (C_2_H_4_) of 42% with good reproducibility. Figure 7 demonstrates that the catalyst Cu_2_O 0.02–1,500 (70°C) has the optimal theoretical electroactive area, and Figure 8 shows the optimal electron transfer rate for the catalyst Cu_2_O 0.02–1,500 (70°C), which are based on the comparison of synthesized catalysts at other temperatures. During 10 h of continuous catalytic use the FE remained essentially undecayed at about 40% as shown in Figure 9.

### 3.3 Catalytic mechanism study

**FIGURE 10:**
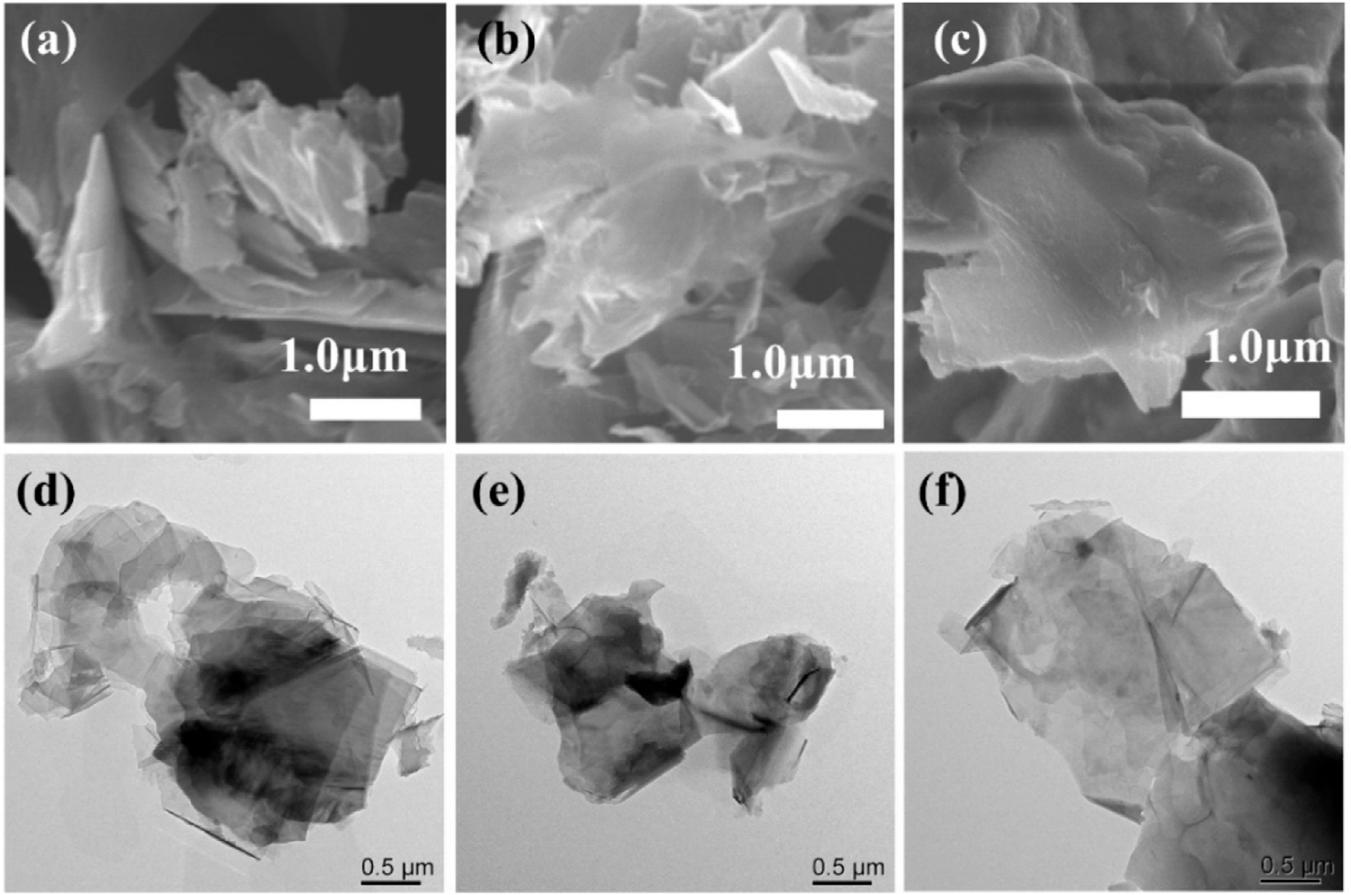
SEM images of **(A)** 0.02–1,500 (60°C), **(B)** Cu_2_O 0.02–1,500 (70°C) and **(C)** Cu_2_O 0.02–1,500 (80°C) catalysts after use. TEM images of **(D)** Cu_2_O 0.02–1,500 (60°C), **(E)** Cu_2_O 0.02–1,500 (70°C) and **(F)** Cu_2_O 0.02–1,500 (80°C) catalysts after use.

**FIGURE 11:**
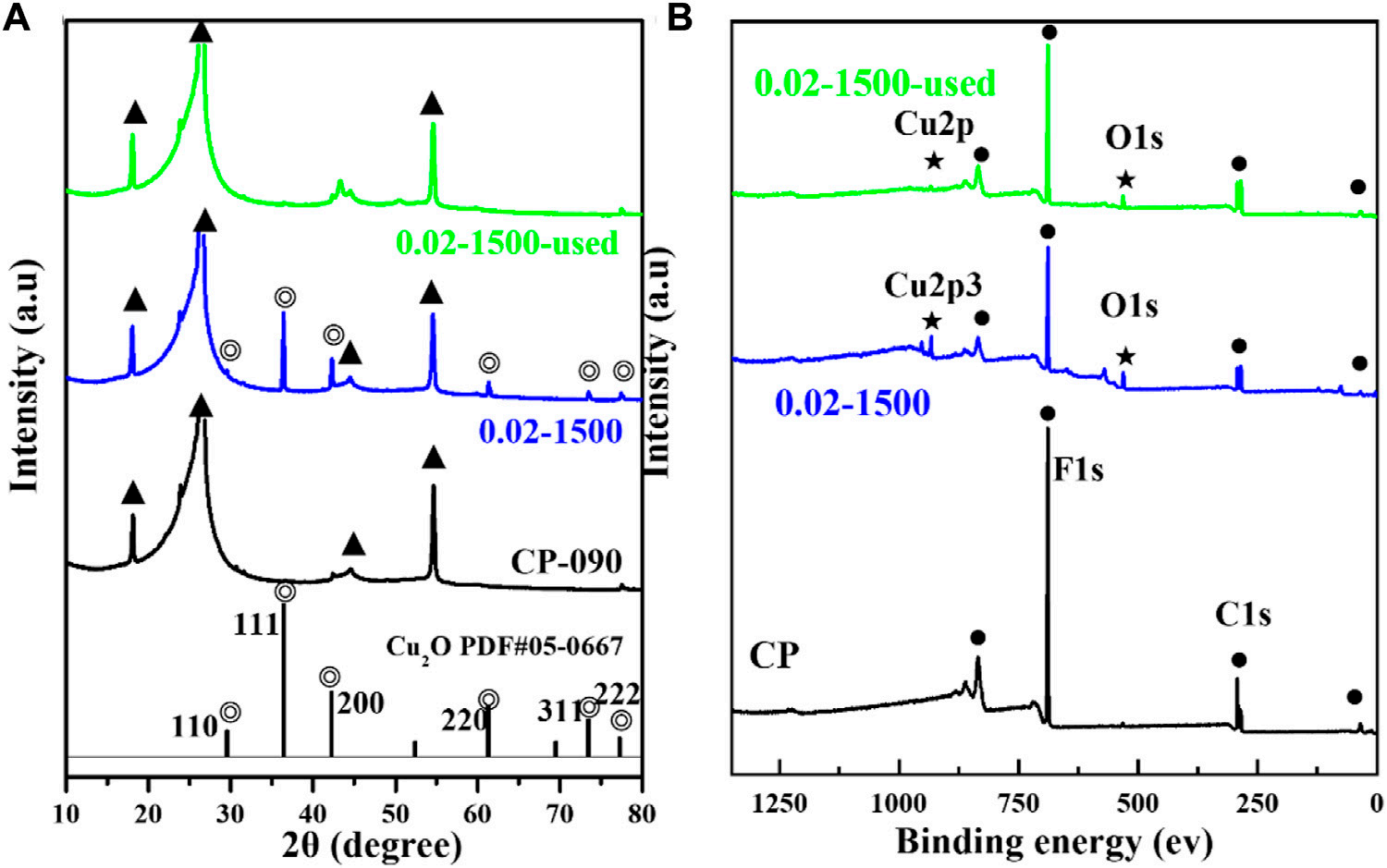
**(A)** Cu_2_O (0.02–1,500), Cu_2_O (0.02–1,500) after being used, and XRD spectra of CP, **(B)** Cu_2_O (0.02–1,500), Cu_2_O (0.02–1,500) after being used, and XPS spectra of CP.

**FIGURE 12:**
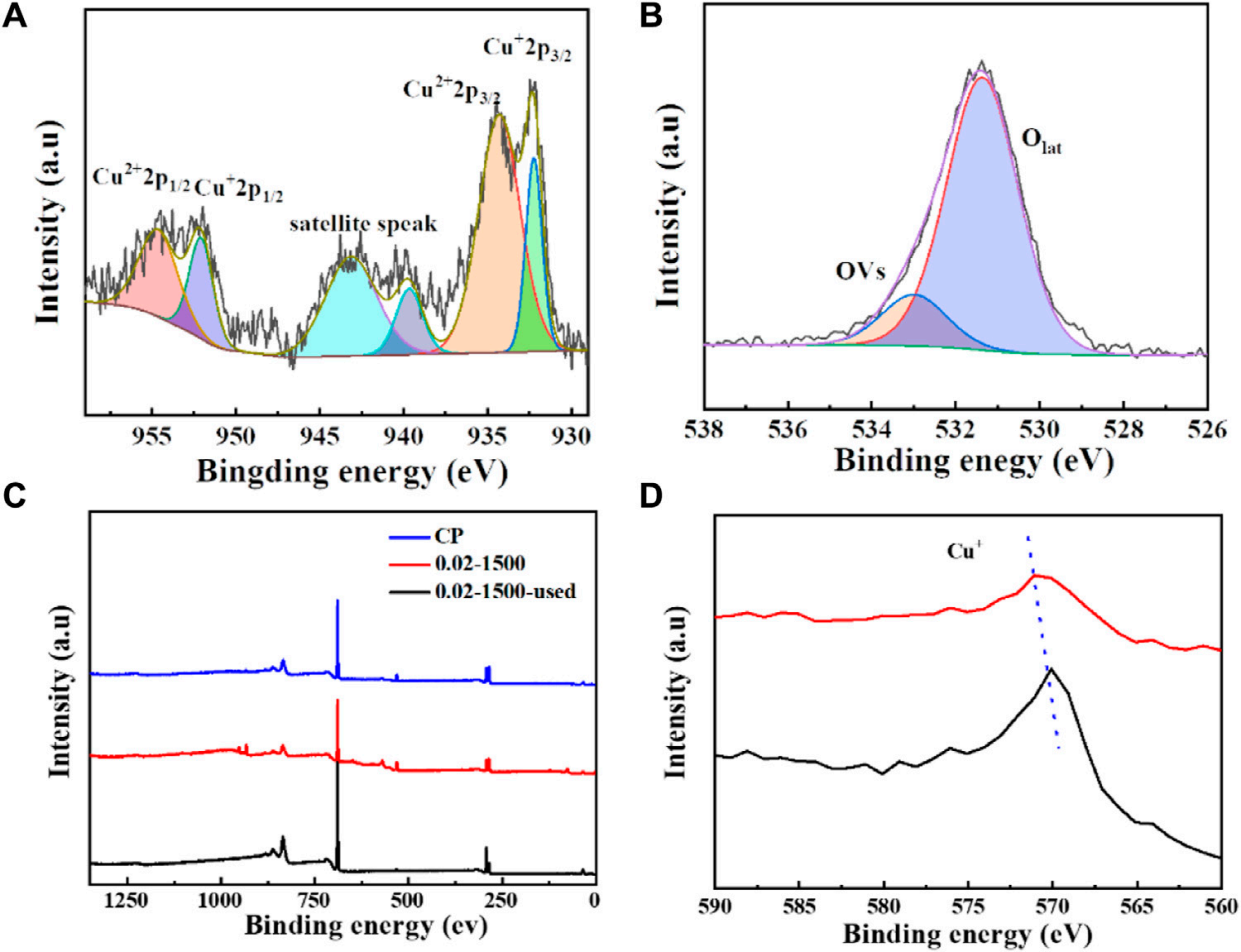
**(A)** 0.02–1,500 (70°C) Cu 2p spectra, **(B)** 0.02–1,500 (70°C) O 1 s spectra, **(C)** CP, 0.02–1,500 (70°C), and 0.02–1,500 (70°C) were used after the spectra XPS spectra **(D)** 0.02–1,500 (70°C) and 0.02–1,500 (70°C) were used Cu LM2 spectra after being used.

We tested the morphology of Cu_2_O catalysts after electrochemical CO_2_ reduction reaction, as shown in Figure 10. The Cu_2_O catalysts after electrolysis failed to maintain the original cross-sectional octahedral morphology. The Cu_2_O catalysts formed nanosheet morphology during the electrolysis process, which may be due to the reduction of Cu_2_O at a more negative potential. Figures 11A, 12C show that many of the original crystalline surfaces of the Cu_2_O microcrystals obviously disappeared after electrochemical CO_2_ reduction, which further proved that Cu_2_O was reduced during the electrochemical reaction. From the XPS spectra in Figures 11B, 12A, it can be seen that the characteristic peak areas of Cu^+^ and Cu^2+^ of the Cu catalyst obtained after being reduced compared with the characteristic peaks of Cu^+^ and Cu^2+^ in the Cu_2_O catalyst (Figure 4A), and the proportion of the peak areas of Cu^+^ 2p^3/2^ and Cu^+^ 2p^1/2^ of the Cu catalyst obtained from the reduced Cu_2_O was reduced, proving that Cu+ and Cu^2+^ in the catalyst were reduced to Cu0 and Cu+. Figure12B depicts the high-resolution O1s spectra of the prepared Cu_2_O:0.02–1,500 (70°C) catalysts after the electrochemical CO_2_ reduction reaction. As shown in Figure 4B, the two characteristic peaks resolved at 529.95 eV and 531.33 eV binding energies are Cu_2_O lattice oxygen (Olat) and oxygen vacancies (OVs), respectively. After the electrochemical CO_2_ reduction reaction, there are oxygen vacancies (OVs) at the binding energy of 531.33 eV, while the Cu_2_O lattice oxygen (O lat) basically disappears, as shown in Figure 12B. The Auger electron spectroscopy (AES) Cu LMM signals of Cu_2_O 0.02–1,500 (70°C) (Figure 12D), at the binding energy of 570.8 eV, show a characteristic peak, which confirms that Cu(I) is the major chemical valence of Cu species. After the electrochemical CO_2_ reduction reaction, a characteristic peak at the binding energy of 570.8 eV is shown, which confirms that Cu (0) is the main chemical valence of the Cu species after derivatization. This proves that the catalyst Cu_2_O is derivatized to Cu^0^ species after electrochemical CO_2_ reduction reaction.

By comparing the CO_2_ reduction performance of Cu_2_O catalysts under different preparation conditions, Cu_2_O 0.02–1,500 (70°C) with regular morphology and the most intact cross-sectional octahedra with exposed crystal faces (111) has a higher selectivity for the conversion of CO_2_ to C_2_H_4_. The complete exposure of crystal faces is particularly important for electrocatalytic conversion of CO_2_ to C_2_H_4_ and is an important factor affecting the increase of FE (C_2_H_4_).C-C coupling is a crucial step in electrocatalytic conversion of CO_2_ to C_2_H_4_, and intermediate adsorption completes the coupling of C-C to C_2_/C_2+_ products. The Cu_2_O 0.02–1,500 (70°C) catalysts with a Cu_2_O 0.02–1,500 (70°C) catalysts with complete morphology and exposed (111) crystal surface are the key active sites for C-C coupling in the catalytic process. The derivatives obtained by a reduction of the catalyst with well exposed crystalline surfaces are the key active sites for the catalytic conversion of CO_2_ into C_2_H_4_ (Gao et al., 2020). During the CO_2_ electroreduction process, *CO is considered to an important intermediate which is further reduced to C_2_H_4_ over Cu_2_O-based catalysts. For C_2_/C_2+_ products, this phenomenon may be attributed to the severe aggregation of *CO on the surface of the catalysts’ Cu_2_O-reduced derivatives promoting further C-C coupling. Cu(I) can be reduced to Cu (0) during the catalytic process, so the center of catalytic activity is viewed as a derivative catalyst with Cu (0). In summary, the active substance in the reduced electrocatalytic conversion of CO_2_ by Cu_2_O is the reduced derived Cu (0).

### 3.4 Conclusion

Cross-sectioned octahedral Cu_2_O microcrystals were prepared *in situ* on carbon paper electrodes by electrochemical deposition. The morphology and integrity of the exposed crystal surface (111) were successfully regulated by controlling the deposition potential, deposition time and deposition temperature. The cross-sectional octahedral Cu_2_O microcrystals have high activity and selectivity for the preparation of C_2_H_4_ by electrocatalytic CO_2_ reduction. The FE (C_2_H_4_) was stabilized at about 40% during 10 h of continuous electrolysis. The cross-sectioned octahedral Cu_2_O microcrystals with intact exposed crystal faces (111) are reduced derived Cu0 during electrolysis, which can effectively promote C-C coupling and may be the main active site for catalyzing the conversion of CO_2_ to C_2_H_4_.

## Data Availability

The original contributions presented in the study are included in the article/supplementary material, further inquiries can be directed to the corresponding author.
